# Incorporating *trnH-psbA* to the core DNA barcodes improves significantly species discrimination within southern African Combretaceae

**DOI:** 10.3897/zookeys.365.5728

**Published:** 2013-12-30

**Authors:** Jephris Gere, Kowiyou Yessoufou, Barnabas H. Daru, Ledile T. Mankga, Olivier Maurin, Michelle van der Bank

**Affiliations:** 1 African Centre for DNA Barcoding, Department of Botany & Plant Biotechnology, University of Johannesburg, PO Box 524, South Africa; 2 C4 EcoSolutions, 9 Mohr Road Tokai, Cape Town, South Africa 7945

**Keywords:** DNA barcoding, closely related species, Combretaceae, southern Africa

## Abstract

Recent studies indicate that the discriminatory power of the core DNA barcodes (*rbc*La + *mat*K) for land plants may have been overestimated since their performance have been tested only on few closely related species. In this study we focused mainly on how the addition of complementary barcodes (nrITS and *trnH-psbA*) to the core barcodes will affect the performance of the core barcodes in discriminating closely related species from family to section levels. In general, we found that the core barcodes performed poorly compared to the various combinations tested. Using multiple criteria, we finally advocated for the use of the core + *trnH-psbA* as potential DNA barcode for the family Combretaceae at least in southern Africa. Our results also indicate that the success of DNA barcoding in discriminating closely related species may be related to evolutionary and possibly the biogeographic histories of the taxonomic group tested.

## Introduction

Combretaceae is a medium-sized family within Myrtales, comprising about 500 species in 17 to 23 genera. It has long been referred to as a complex phylogenetic and taxonomic group (Tan et al. 2002, Maurin et al. 2010, Stace 2010, Jordaan et al. 2011). Based on morphological characters and phylogenetic analysis, the family Combretaceae has been recovered as monophyletic and sister to the rest of Myrtales (Brown 1810, Dahlgren and Thorne 1984, Tan et al. 2002, Sytsma et al. 2004, Maurin et al. 2010, Stace 2010). Members of Combretaceae are mainly trees, shrubs or lianas, occupying a wide range of habitats from savannas, forests, to woodlands (Maurin et al. 2010) and are distributed in tropical and subtropical regions across the globe. With ca. 350 species, *Combretum* Loefl., the largest genus in the family has its centre of diversity in Africa, with approximately 63 species described in southern Africa – south of the Zambezi river and includes South Africa, Zimbabwe, Namibia, Botswana, Lesotho, Swaziland, and Mozambique (Maurin et al. 2010, Jordaan et al. 2011).

**Figure 1.**
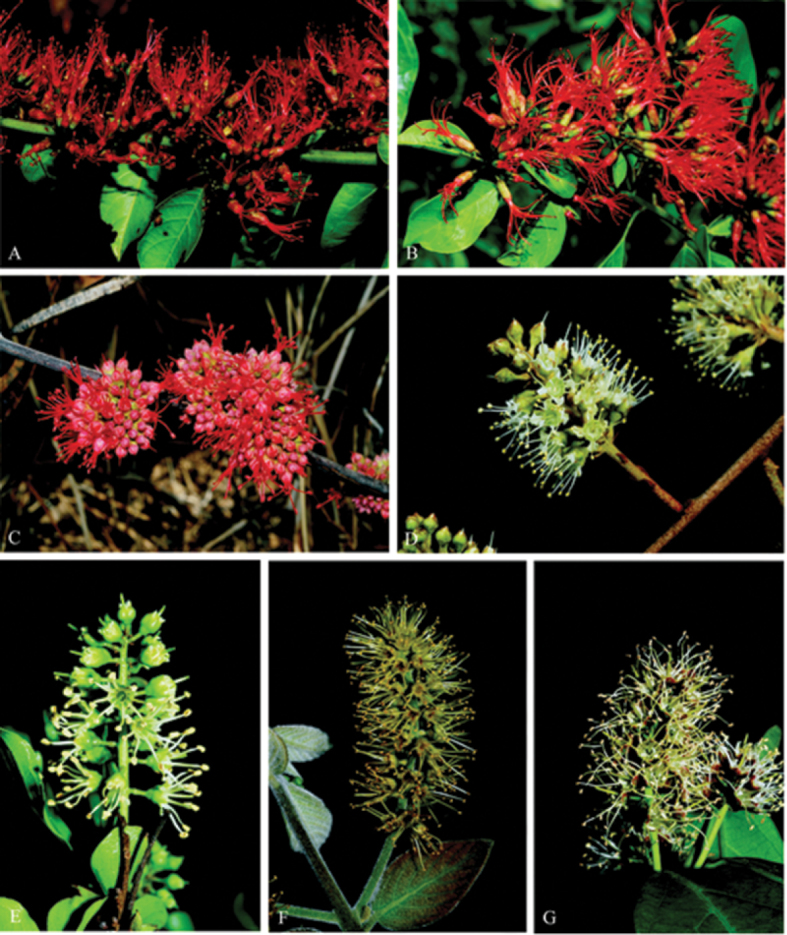
Selected inflorescences of seven *Combretum* species indicating closely related species evaluated based upon floral characters. **A** *Combretum paniculatum* **B** *Combretum microphyllum* **C** *Combretum platypetalum* **D** *Combretum hereroense* **E** *Combretum apiculatum* **F** *Combretum molle* **G** *Combretum kraussii*. All photographs by O. Maurin.

**Figure 2.**
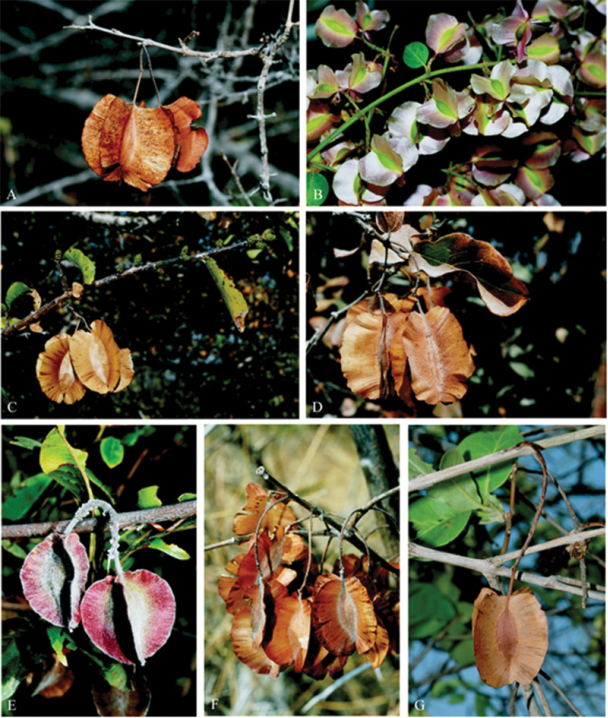
Selectedmature dry four-winged fruits of closely related species of genus *Combretum*. **A** *Combretum mkuzense* **B** *Combretum microphyllum* **C** *Combretum englerii* **D** *Combretum apiculatum* **E** *Combretum moggii* **F** *Combretum albopunctatum* **G** *Combretum collinum*. All photographs by O. Maurin.

The major distinguishing feature of the family is the presence of unicellular combretaceous hairs on the abaxial leaf surfaces, a diagnostic trait in many other species of Myrtales and even beyond the group e.g. the Cistaceae Juss. family, tribe Cisteae (Maurin et al. 2010, Stace 2010). However, other morphological features such as presence of trichomes, stalked glands, domatia, inflorescence, fruit shape, leaf and pollen morphology are also important for species delimitation in Combretaceae (Exell and Stace 1966, Stace 2007, 2010, Maurin et al. 2010, Jordaan et al. 2011). Nonetheless, all these characters are not adequate enough to delimit species within the family because none is unique to a specific clade. As a result, the family has experienced several splitting and lumping in the past (El Ghazlai et al. 1998, Tan et al. 2002, Maurin et al. 2010, Stace 2010, Jordaan et al. 2011). Also, the taxonomy is further confounded by the high morphological similarity between members of different sections. For instance, inflorescence and fruit shapes are very similar between species and across clades (Figures 1 and 2). Such homoplasious morphological similarities have also been identified as the root of difficulties in delimiting the genera; for example in the *Combretum*-*Quisqualis* clade (Jordaan et al. 2011). Consequently, it becomes necessary to search for an alternative method to augment traditional morphology-based taxonomy of Combretaceae.

Here, we propose that DNA barcoding may provide such a complementary tool to ease species delimitation within the group. DNA barcoding involves the use of a short and standardised DNA sequence that can help assign, even biological specimens devoid of diagnostic features, to species (Hebert et al. 2004, 2010, Hajibabaei et al. 2006, Roy et al. 2010, Van der Bank et al. 2012, Franzini et al. 2013). Two DNA regions defined as ‘core barcodes’, i.e. *rbc*La and *mat*K have been standardised as DNA barcodes for land plants (CBOL Plant Working Group 2009). In addition to the core barcodes, two other regions, *trnH-psbA* and nrITS were suggested as supplementary DNA barcodes for plants (Hollingsworth et al. 2011, Li et al. 2011). The rationale for adopting these two regions (*rbc*La and *mat*K) is high levels of recoverability of high-quality sequences and acceptable levels of species discrimination (Burgess et al. 2011). The discriminatory power of the core DNA barcodes for land plants was estimated at 70–80% (CBOL Plant Working Group 2009, Fazekas et al. 2009, Kress and Erickson 2007). However, a recent study suggests that efficacy of core barcodes may have been overestimated, arguing that taxon sampling has been biased towards less-related species (Clement and Donoghue 2012). Furthermore, barcoding efficacy is rarely evaluated in a phylogenetic context (but see Clement and Donoghue 2012), resulting in potentially biased estimates of discriminatory power.

In this study, we evaluated the efficacy of DNA barcoding as a tool to augment morphological species discrimination within Combretaceae. Specifically, we (1) assessed the potential of four markers to discriminate southern African species of the family, and (2) assessed the efficacy of barcodes across major clades including subgenera and sections within the largest genus *Combretum*.

## Methods

Sampling includes one to six accessions of 58 species out of the 63 species representing the six genera of Combretaceae in southern Africa. These genera include *Combretum* (43 species included in this study), *Lumnitzeria* Wild. (one species included), *Meiostemon* Exell and Stace (one species included), and *Quisqualis* L. (one species included), *Pteleopsis* Engl. (two species included), and *Terminalia* (ninespecies included).

Collection details, taxonomy, voucher numbers, GPS coordinates, field pictures, and sequence data (only *mat*K and *rbc*La) are archived online on the BOLD system (www.boldsystems.org). Voucher information, name of herbarium, GenBank and BOLD accession numbers are listed in Appendix 1.

### DNA extraction, amplification and alignment

Genomic DNA was extracted from silica gel-dried and herbarium leaf material following a modified cetyltrimethyl ammonium bromide (CTAB) method of Doyle and Doyle (1987). To ease the effects of high polysaccharide concentrations in the DNA samples, we added polyvinyl pyrolidone (2% PVP). Purification of samples was done using QIAquick purification columns (Qiagen, Inc, Hilden, Germany) following the manufacturer’s protocol.

All PCR reactions were carried out using Ready Master Mix (Advanced Biotechnologies, Epsom, Surrey, UK). We added 4.5% of dimethyl sulfoxide (DMSO) to the PCR reactions of nrITS to improve PCR efficiency. Amplification of *rbc*La was done using the primer combination: 1F: 724R (Olmstead et al. 1992, Fay et al. 1998). For *mat*K, the following primer combination was used 390F: 1326R (Cuénoud et al. 2002). Intergenic spacers *trnH-psbA* and *psaA-ycf3* were amplified using the primers *trnH*: *psbA* (Sang et al. 1997) and PG1F: PG2R (Huang and Shi 2002), respectively. Intergenic spacer *psaA*-*ycf3* was included in this study for the purpose of reconstructing phylogeny of Combretaceae. The nrITS region was amplified into two overlapping fragments using the following two pairs of internal primer combinations: 101F: 2R and 3F: 102R (White et al. 1990, Sun et al. 1994).

The following programme was used to amplify *rbc*La and *trnH-psbA*: pre-melt at 94 °C for 60 sec, denaturation at 94 °C for 60 sec, annealing at 48 °C for 60 sec, extension at 72 °C for 60 sec (for 28 cycles), followed by a final extension at 72 °C for 7 min; for *mat*K, the protocol consisted of pre-melt at 94 °C for 3 min, denaturation at 94 °C for 60 sec, annealing at 52 °C for 60 sec, extension at 72 °C for 2 min (for 30 cycles), final extension at 72 °C for 7 min. For nrITS and spacer *psaA-ycf3* the protocol consisted of pre-melt at 94 °C for 1 min, denaturation at 94 °C for 60 sec, annealing at 48 °C for 60 sec, extension at 72 °C for 3 min (for 26 cycles), final extension at 72 °C for 7 min.

Purification of the amplified products was done using QIAquick columns (QIAgen, Germany) following the manufacturer’s manual. The purified products were then cycle-sequenced with the same primers used for amplification using BigDye^TM^ v3.1 Terminator Mix (Applied Biosystems, Inc, ABI, Warrington, Cheshire, UK). Cleaning of cycle-sequenced products was done using EtOH-NaCl, followed by sequencing on an ABI 3130xl genetic analyser.

Sequences were assembled, trimmed and edited using Sequencher v4.6 (Gene Codes Corp, Ann Arbor, Michigan, USA). Alignment was done using Multiple Sequence Comparison by Log-Expectation v3.8.31 (Edgar 2004) followed by subsequent manual adjustments to refine alignments.

### Data analysis

Performance of DNA markers in species delimitation was tested at three taxonomic levels (family, subgenus, and section). At family level, we evaluated four single markers: *rbc*La, *mat*K, *trnH-psbA*, and nrITS. We also tested the core barcodes, i.e. *rbc*La + *mat*K (CBOL Plant Working Group 2009) and the following combinations: core + nrITS, core + *trnH-psbA*, and core + *trnH-psbA* + nrITS. Four criteria were used to assess their barcoding potential: presence of ‘barcode gap’ (Meyer and Paulay 2005), discriminatory power, species monophyly, and PCR success rate.

Barcode gap was evaluated in two ways: (1) we compared genetic variation within species (intraspecific genetic distance) versus between species (interspecific genetic distance). This comparison was based on the mean, median, and range of both distances; (2) in addition, we also used Meier et al.’s (2008) approach of evaluating the gap comparing the smallest interspecific distance with the greatest intraspecific distance. The genetic distances were calculated using the Kimura 2-parameter (K2P) model. We also assessed the index of sequence divergence, K, for each region, measured as the mean number of substitutions between any two sequences.

The discriminatory power of DNA regions was conducted using three distance-based methods including Near Neighbour, Best Close Match (Meier et al. 2006) and the BOLD identification criteria. A good barcode should exhibit the highest rate of correct species identification by assigning the highest proportion of DNA sequences to the corresponding species names. All the sequences were labelled according to species names prior to testing. For the Best Close Match test, we determined, for each dataset (family, subgenera and sections), the optimised genetic distance suitable as threshold for species delimitation. Optimised thresholds were determined using the function “localMinima” implemented in the R package Spider v1.1-1 (Brown et al. 2012).

We also used the PCR success rate to evaluate the DNA regions. This evaluation was conducted based on the percentage of successful amplification.

The test for species monophyly was conducted on a Neighbour-Joining (NJ) tree. We considered that a species is monophyletic when all individuals of the same species cluster on the NJ phylogram that we reconstructed. As such, the best barcode should provide the highest proportion of monophyletic species. We then evaluated for each DNA region and concatenated regions, the proportion of monophyletic (i.e. correct identification) and non-monophyletic species (incorrect identification). All our analyses were conducted in the R package Spider v1.1-1 (Brown et al. 2012).

Finally, we evaluated the barcoding potential in discriminating phylogenetically deliminated clades in the phylogeny of the genus that was reconstructed based on the combination of five DNA regions (*rbc*L, *mat*K, *trnH-psbA*, *psaA-ycf3* and nrITS). The phylogeny was reconstructed based on maximum parsimony (MP) implemented in PAUP* v4.0b10 (Swofford 2002). Tree searches were conducted using heuristic searches with 1 000 random sequence additions, retaining 10 trees per replicate, with tree-bisection-reconnection (TBR) branch swapping and MulTrees in effect (saving multiple equally parsimonious trees). Based on Maurin et al. (2010) we used *Strephonema mannii* Hook. f. and *Strephonema pseudocola* A. Chev. as outgroups. Node support was assessed using bootstrap (BP) values: BP > 70% for strong support (Hillis and Bull 1993, Wilcox et al. 2002).

At subgeneric and sectional levels, we only tested the performance of core barcodes and best gene combination identified using the three criteria mentioned above (barcode gap, discriminatory power and species monophyly).

## Results

The overall characteristics of single and combined DNA regions are presented in Table 1. In general, our results indicate that the ranges and mean intraspecific distances were both lower than those of interspecific distances. Among single regions, *rbc*La showed the lowest interspecific distance (mean = 0.009) with nrITS exhibiting the highest genetic variation between species (mean = 0.110). For all marker combinations, the mean interspecific distances varied between 0.011 and 0.014. Assessing the index of sequence divergence K for each region, we found that nrITS showed the highest divergence (K = 21) whereas *trnH-psbA* exhibited the lowest divergence (K = 3). For the combined regions, K varied between 10 and 13, with an average of 10 substitutions between sequence-pairs (Table 1).

**Table 1.** Statistics of all gene regions for the southern African Combretaceae included in the study.

DNA regions	No. of seq	Seq length	K	Range inter	Mean inter (±SD)	Range intra	Mean intra (±SD)	Threshold (%)
*rbc*La	152	552	4	0-0.09	0.009±0.012	0-0.08	0.002±0.009	0.04
*mat*K	133	771	6	0-0.07	0.014±0.011	0-0.02	0.002±0.004	1.10
*trnH-psbA*	116	1034	3	0-0.15	0.047±0.035	0-0.03	0.003±0.007	1.80
nrITS	91	821	21	0-0.21	0.110±0.045	0-0.05	0.004±0.010	1.70
*rbc*La+*mat*K	129	1323	10	0-0.78	0.012±0.009	0-0.05	0.002±0.006	1.31
*rbc*La+*mat*K+*trnH-psbA*	87	2358	11	0-0.04	0.012±0.007	0-0.02	0.002±0.004	0.5
*rbc*La+*mat*K+nrITS	74	2144	9	0-0.04	0.011±0.006	0-0.02	0.002±0.004	0.70
*rbc*La+*mat*K+nrITS+*trnH-psbA*	70	3178	13	0-0.04	0.014±0.007	0-0.02	0.002±0.004	1.17

**Figure 3.**
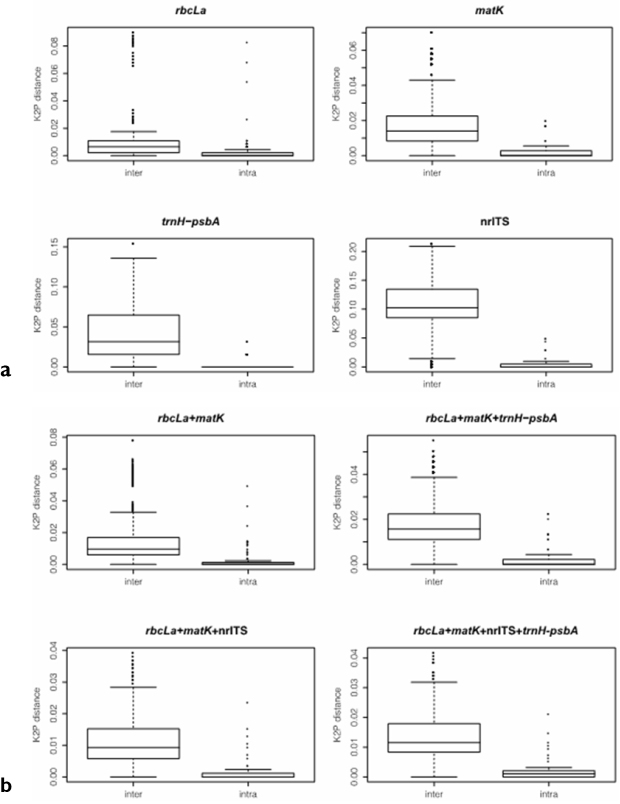
Comparisons of the distribution range of inter- versus intraspecific distances using boxplot **a** indicates comparison of single barcode gene regions **b** indicates the results of gene combinations.

**Figure 4.**
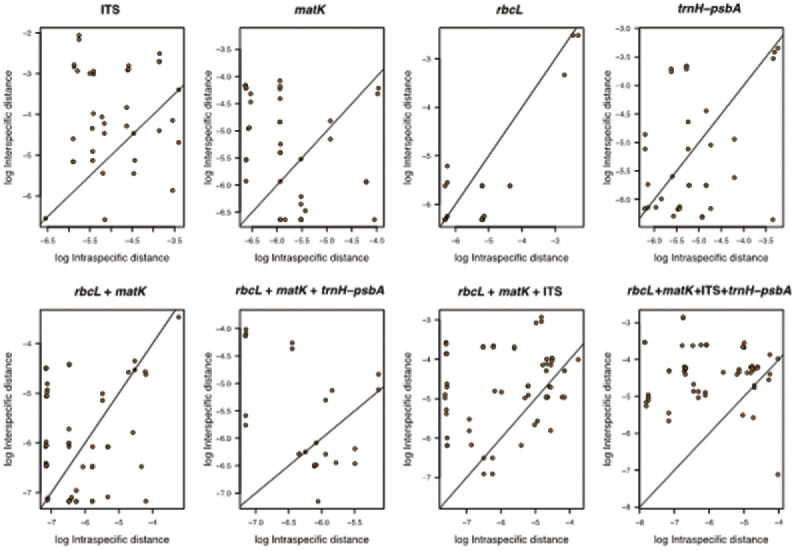
Relationships between inter- and intraspecific distances indicating barcoding gap for all regions tested.

The distribution ranges of inter- versus intraspecific distances for all regions, showed a clear overlap between both distances (Figures 3a, b and 4), indicating the existence of a barcode gap. Comparing the smaller inter- versus the largest intraspecific distances for each region, our results further support the existence of barcode gap in all regions, but the proportion of sequences with barcode gap varied significantly with the regions tested (Table 2). Notably, the combination of all four regions exhibited the highest proportion of sequences with barcode gap (84%) followed by nrITS (73%), then core + nrITS (64%), and core + *trnH-psbA* (57%), with the lowest proportion found in *rbc*La (13%) (Table 2).

Optimised genetic distances used as threshold for species delimitation in Best Close Match method are shown in Table 1. Apart from *rbc*La (threshold = 0.04%), core + *trnH-psbA* (threshold = 0.5%) and core + nrITS (threshold = 0.7%), the thresholds for the remaining single and gene combinations were greater than 1%.

**Table 2.** Percentage barcode gap in all sequences for each region using the Meier et al. (2008) approach.

DNA region	Number of sequences without gap	Proportion of sequences with gap (%)
*rbc*La	132	13
*mat*K	86	35
*trnH-psbA*	54	53
nrITS	25	73
*rbc*La+*mat*K	82	36
*rbc*La+*mat*K+*trnH-psbA*	37	57
*rbc*La+*mat*K+nrITS	27	64
*rbc*La+*mat*K+nrITS+*trnH-psbA*	11	84

Our results for the discriminatory power analysis varied with the methods applied (Table 3) at family level. Based on the Near Neighbour method, nrITS provided the highest discriminatory power (65%) followed by *rbc*La + *mat*K + *trnH-psbA* + nrITS (64%), *rbc*La + *mat*K + *trnH-psbA* (62%), and *rbc*La + *mat*K (61%). The lowest discriminatory power was found for *trnH-psbA* (28%).

**Table 3.** Identification efficacy of DNA barcodes using distance based methods. F = False and T = True.

DNA region	Near Neighbour		BOLD (1%)				Best Close Match			
	F (%)	T (%)	Ambiguous (%)	Correct (%)	Incorrect (%)	No ID (%)	Ambiguous (%)	Correct (%)	Incorrect (%)	No ID (%)
*rbc*La	59	41	61	18	14	7	61	18	14	7
*mat*K	46	54	81	11	7	1	47	38	14	1
*trnH-psbA*	72	28	65	22	10	3	18	60	18	4
nrITS	35	65	29	47	10	14	10	63	19	8
*rbc*La+*mat*K	39	61	86	10	2	2	35	51	12	2
*rbc*La+*mat*K+*trnH-psbA*	38	62	79	16	2	3	6	80	8	6
*rbc*La+*mat*K+nrITS	43	57	62	30	7	1	3	70	19	8
*rbc*La+*mat*K+nrITS+*trnH-psbA*	36	64	52	41	3	4	0	87	9	4

BOLD species delimitation criteria of 1% threshold provided the lowest rate of correct identification among all three methods used. However, we found that nrITS remains the most efficient region with 47% discriminatory power. The second most successful combination of regions were core + *trnH-psbA* + nrITS (41%) followed by core + nrITS (30%) and *trnH-psbA* (22%); the core barcodes were identified as the least performing regions (10%) with the highest proportion of ambiguity (86%).

In contrast to the two previous methods, the Best Close Match provided the highest rate of species discrimination for the combined dataset (core + *trnH-psbA* + nrITS) yielding the best discriminatory power (87%) with no ambiguity. This was followed by core + *trnH-psbA* (80%), core + nrITS (70%) and nrITS (63%), with the poorest performance for *rbc*La (18%) at family level.

**Figure 5.**
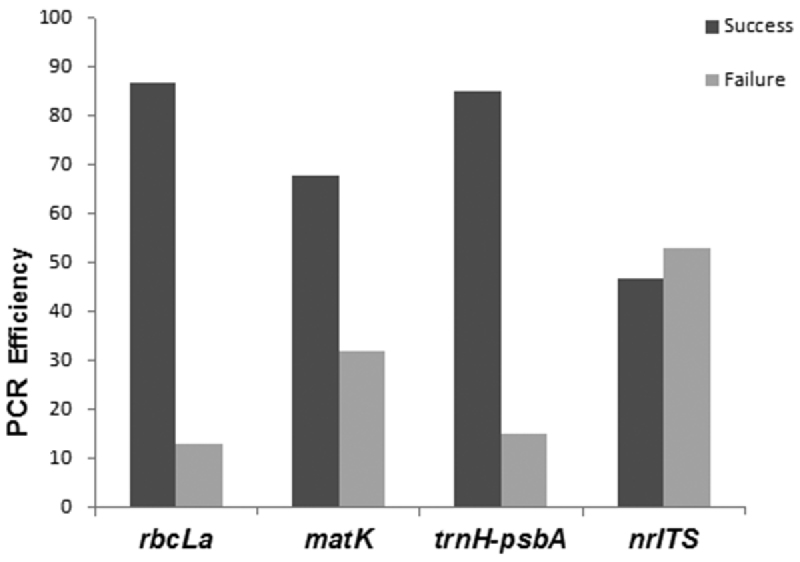
PCR efficiency for the four candidate barcodes (*rbc*La, *mat*K, *trnH-psbA*, nrITS).

The last criterion used to evaluate the potential of DNA region was PCR efficiency. We found that *rbc*La (87%) followed by *trnH-psbA* (85%) and *mat*K (68%) were easy to amplify, with nrITS being the most difficult (47%; Figure 5).

**Figure 6.**
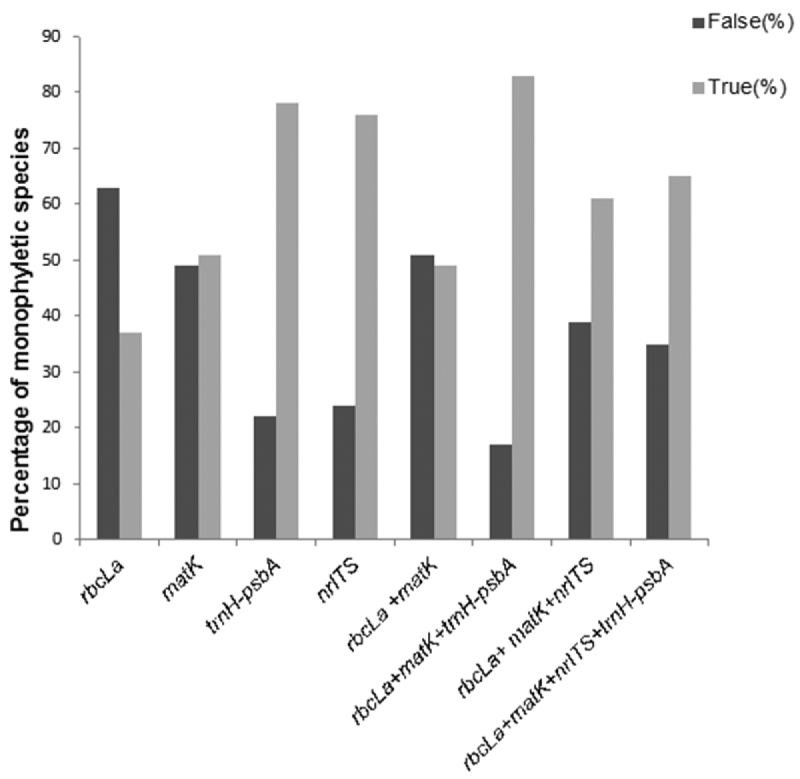
Gene performance based on monophyly criteria. False = proportion of non-monophyletic species; True = proportion of monophyletic species.

We complemented previous analyses using species monophyly criteria after verifying the monophyly of Combretaceae. Among all regions, core + *trnH-psbA* isolated the highest proportion of monophyletic species (83%), followed by *trnH-psbA* (78%), nrITS (76%), and combination of all four regions (65%). Again, *rbc*La provided the lowest performance in identifying species as monophyletic (37%; Figure 6).

In summary, all regions provided evidence for barcode gaps (Figure 3a, b and 4), but the strength of evidence varied with approaches used. Furthermore, the Best Close Match method provided the highest identification accuracy among the three distance-based methods used irrespective of genes or combinations tested. Under this method, the two best potential barcodes for southern African Combretaceae were first, core+ *trnH-psbA* and second, core + *trnH-psbA* + nrITS. However, based on species monophyly criteria, the single region *trnH-psbA* and the combination core + *trnH-psbA* showed high barcode potential, with *trnH-psbA* being the second best easy-to-amplify region after *rbc*La.

We further evaluated the potential of each region as candidate barcode using a phylogeny of southern African Combretaceae (Appendix 2). Our results are congruent to the corresponding subset in the most recent and largest phylogeny assembled for the family (Appendix 3). Our evaluation for the discriminatory power at subgeneric level using the thresholds determined for the family (1.31% for the core and 0.5% for the core + *trnH-psbA*) revealed that the core barcodes alone were able to correctly identify 78% of species within the subgenus *Cacoucia*. However, the core barcodes could discriminate only 50% of species within the subgenus *Combretum*. In particular, the discriminatory power of the core barcodes within both subgenera increased markedly to 100% when we added the *trnH-psbA* region (Table 4). This trend was consistent even when we applied the thresholds that have been optimised for the subgenera.

At sectional level, we observed similar trends – the addition of *trnH-psbA* increased the performances of the core barcodes drastically except for *Macrostigmatea* (Table 5): *Angustimarginata* (core: 11%; core + *trnH-psbA*: 86%); *Ciliatipetala* (core: 55%; core + *trnH-psbA*: 73%); *Conniventia* (core: 38%; core + *trnH-psbA*: 88%); *Hypocrateropsis* (core: 63%; core + *trnH-psbA*: 80%). However, *Macrostigmatea* (core 34%, core + *trnH-psbA* 44%) showed the least performance, even with the addition of *trnH-psbA* to the core barcode, with just 10% increment being observed. This trend is not sensitive to the thresholds applied for the family or the sections.

**Table 4.** Comparisons of efficacy of core barcodes and best barcode within subgenera *Combretum* and *Cacoucia*.

Subgenus	DNA region	No. of seq	Mean Inter (±SD)	Threshold (%)	Best Close Match			
					Ambiguous (%)	Correct (%)	Incorrect (%)	No ID (%)
*Cacoucia*	*rbc*La+*mat*K	23	0.004±0.002	1.31	13	78	9	0
	*rbc*La+*mat*K+*trnH-psbA*	16	0.006±0.002	0.5	0	100	0	0
*Combretum*	*rbc*La+*mat*K	84	0.009±0.009	1.31	36	50	12	2
	*rbc*La+*mat*K+*trnH-psbA*	16	0.006±0.002	0.5	0	100	0	0

**Table 5.** Comparisons of core barcodes and the best barcode within five sections of the subgenera *Combretum* and *Cacoucia*.

Sections	DNA regions	No. of seq	Mean inter (±SD)	Threshold (%)	Best Close Match			
					Ambiguous (%)	Correct (%)	Incorrect (%)	No ID (%)
*Angustimarginata*	*rbc*La+*mat*K	19	0.007±0.014	2.6	58	11	26	5
	*rbc*La+*mat*K+*trnH-psbA*	15	0.006±0.006	*0.7*	0	86	7	7
*Ciliatipetala*	*rbc*La+*mat*K	20	0.004±0.002	0.3	45	55	0	0
	*rbc*La+*mat*K+*trnH-psbA*	15	0.006±0.003	*0.5*	0	73	27	0
*Conniventia*	*rbc*La+*mat*K	8	0.005±0.004	0.8	37	38	12	13
	*rbc*La+*mat*K+*trnH-psbA*	8	0.010±0.006	*2.4*	*0*	88	12	0
*Hypocrateropsis*	*rbc*La+*mat*K	8	0.012±0.005	1.31	25	63	12	0
	*rbc*La+*mat*K+*trnH-psbA*	5	0.020±0.004	*0.8*	*0*	80	20	0
*Macrostigmatea*	*rbc*La+*mat*K	15	0.002±0.001	0.1	53	34	13	0
	*rbc*La+*mat*K+*trnH-psbA*	9	0.003±0.002	*0.2*	*0*	44	56	0

(Only sections with at least three different species are included).

Finally, we compared the mean number of substitutions between any two species within each section. We found that the mean number of substitutions between representatives of *Macrostigmatea* is lowest (mean = 4) whereas it ranges between 5 and 19 substitutions in other sections of subgenus *Combretum*.

## Discussion

We evaluated genetic variation for both single and various combinations of *rbc*La, *matK*, *trnH-psbA* and nrITS. Comparing ranges of intra- versus interspecific distances, our results indicate that all markers show a barcode gap (Meyer and Paulay 2005); and this is also true for the stringent Meier et al.’s (2008) approach, although the proportion of sequences with gap varies greatly with the marker used.

The discriminatory power of the DNA regions in species identification also varies with the distance-based methods applied. From the methods tested, Near Neighbour and Best Close Match yielded high performance, with the latter giving the best results for the possible three and four different gene combinations. The core barcodes were not recognised among the three best options, and its discriminatory power has been questioned in a number of studies (Hollingsworth et al. 2009, Pettengill and Neel 2010, Roy et al. 2010, Wang et al. 2010, Clement and Donoghue 2012). Based on all three distance methods, nrITS emerges as the most suitable single region (as indicated under both Near Neighbour and BOLD; see also Kress et al. 2005, Kress and Erickson 2007, Chen et al. 2010, Gao et al. 2010, Ren et al. 2010, China Plant BOL Group et al. 2011, Muellner et al. 2011, Pang et al. 2011, Wang et al. 2011, Liu et al. 2012, Yang et al. 2012). Among combined regions, core + nrITS + *trnH-psbA* (under Best Close Match) emerges as most suitable for barcoding Combretaceae.

However, our study indicates some important drawbacks that discount the inclusion of nrITS as a good barcode. For example, based on amplification success criteria, nrITS was the most difficult of all regions tested with *rbc*La and *trnH-psbA* being the easiest regions to amplify. The technical hurdles in PCR amplification and sequencing of nrITS may be linked to the presence of retro-transposons and other repetitive elements within plant nuclear genomes, resulting in paralogous gene copies (Gao et al. 2010, Hollingsworth 2011, Hollingsworth et al. 2011, Li et al. 2011). This is likely the case for nrITS in Combretaceae as we found evidence of multiple copies that may not be identical to each other (see CBOL Plant Working Group 2009, Hollingsworth 2011, Hollingsworth et al. 2011, Yang and Berry 2011). As such, the addition of *trnH-psbA* to the core barcodes (*rbc*La + *mat*K + *trnH-psbA*) emerge as the best gene combination useful for species discovery and delimitation in Combretaceae (see also Newmaster and Ragupathy 2009, Petit and Excoffier 2009, Ragupathy et al. 2009, Wang et al. 2009, Arca et al. 2012).

Previous studies have shown that core barcodes are very limited in discriminating taxa that are phylogenetically closely related, and suggested that the efficacy of DNA barcodes should be tested within a phylogenetic context (Clement and Donoghue 2012). We tested this using subgenera and sections of the family Combretaceae. Our evaluation of the discriminatory power of the core barcodes at subgeneric level revealed a striking difference in the performance between the two *Combretum* subgenera, *Combretum* and *Cacoucia*. The difference noted for the discriminatory power of the core barcodes between the two subgenera may reflect differences in their evolutionary history. Indeed, the latest dated phylogeny of Combretaceae indicated that members of the subgenus *Cacoucia* are represented with longer terminal branches than those in subgenus *Combretum* (Maurin 2009).

While we found poor performance at sectional level, for example, in *Angustimarginata*, *Macrostigmatea* and *Conniventia*, this result is not unexpected due to a very low genetic variation one could expect within clades (see Ennos et al. 2005, Clement and Donoghue 2012). However, the addition of *trnH-psbA* to the core barcodes results in a drastic increase of identification rate at both subgenus and sectional levels, validating the utility of *trnH-psbA* to discriminate even closely related species, except for section *Macrostigmatea* (Newmaster and Ragupathy 2009, Petit and Excoffier 2009, Ragupathy et al. 2009, Wang et al. 2009, but see Zhang et al. 2012, Arca et al. 2012, Clement and Donoghue 2012).

The result for section *Macrostigmatea* reflects earlier tangle cited in previous studies regarding its composition (Stace 1980, Maurin et al. 2010, Jordaan et al. 2011). In our analysis, we included *Spathulipetala* within section *Macrostigmatea* based on suggestions from recent molecular evidence (Maurin et al. 2010). Morphological studies separate these two sections, *Spathulipetala* and *Macrostigmatea* (Stace 1980, Jordaan et al. 2011). Section *Spathulipetala* comprises two members, *Combretum zeyheri* Sond. and *Combretum mkuzense* J.D.Carr and Retief, which occur in the same geographical location and show close morphological similarity in their fruits (Jordaan et al. 2011). The inclusion of *Combretum mkuzense*, in this section has been controversial, with some authors (Exell 1978, Stace 1980) advocating for a tentative placement pending further investigation. However, recent molecular study shows close relationship between these two species (*Combretum zeyheri* and *Combretum mkuzense*) (Maurin et al. 2010), which gives support to earlier morphological treatment. On the other hand, the taxonomy of section *Macrostigmatea* appears to pose fewer challenges as compared to *Spathulipetala*. A recent molecular study (Maurin et al. 2010) suggests lumping of these two sections, *Spathulipetala* and *Macrostigmatea* as members appear embedded in one clade with a high bootstrap support of 100%. Earlier, Exell (1978) had reported that the sections are closely related, as they share similarities in scale size, scale fragmentation into fruit walls and fruit size.

Based on our results, the unclear taxonomy reported for section *Macrostigmatea*, is reflected, indicating a need for further molecular analyses involving more taxa and gene sequences to correctly determinemembers of this section. Our results also support the proposal of Exell (1978) to lump these two sections. The low performance of the core + *trnH-psbA* in fully discriminating the different species within this section is a strong indicator of the close phylogenetic similarity of the species. Our results indicate not only the utility of DNA barcoding data for discriminating species, but also to detect species that require further molecular analyses.

## Conclusions

Our analysis indicates that the poor performance of the core barcodes at family level could not be generalised to lower levels: the core barcodes perform poorly in some sections but shows strong discriminatory power in others. Such findings may indicate that the success of DNA barcodes in discriminating closely related species at least in plants may correlate with the evolutionary distinctiveness of the group tested and, as recently indicated, (see Clement and Donoghue 2012) it may also possibly reflects different biogeographic history between clades of the taxonomic group Combretaceae. Overall, we propose the core + *trnH-psbA* as the best barcode for the family Combretaceae.

## Appendix 1

Supplementary table S1. (doi: 10.3897/zookeys.365.5728.app1) File format: Microsoft Excel file (xls).

**Explanation note:** Full names, voucher information, GenBank and BOLD accession numbers for taxa used in this study. A dash (—) indicates DNA regions not sampled and DNA sequences obtained from GenBank are underlined. Voucher specimens are deposited in the following herbaria: JRAU, University of Johannesburg (UJ), Johannesburg, South Africa; MO, Missouri Botanical Garden, St Louis, USA.

## Appendix 2

Supplementary figure S1. (doi: 10.3897/zookeys.365.5728.app2) File format: Microsoft Word file (docx).

**Explanation note:** One of most parsimonious trees obtained from the combined plastid and nuclear data (*rbc*La, *mat*K, *trnH-psbA*, and nrITS) set. Clades highlighted indicate the sections that were identified from the MP tree obtained from barcoding gene regions. Bootstrap percentages above 50% are shown above the branches.

## Appendix 3

Supplementary figure S2. (doi: 10.3897/zookeys.365.5728.app3) File format: Microsoft Word file (docx).

**Explanation note:** One of most parsimonious trees with branch tips collapsed from the combined plastid and nuclear data (*rbc*L, *mat*K, *psaA-ycf3*, *trnH-psbA*, and nrITS) set. Clades highlighted indicate sections that were identified from the MP tree obtained from barcoding gene regions. Above the branches are Bayesian posterior probability (PP) values (> 0.5) and below are bootstrap percentages above 50%.
