# Supplementary material for: Incorporating trnH-psbA to the core DNA barcodes improves significantly species discrimination within southern African Combretaceae
Source: Zookeys. 2013 Dec 30;(365):129–47. doi: 10.3897/zookeys.365.5728 (PMC3890675; doi:10.3897/zookeys.365.5728)
Supplement: Supplementary file 3 — Supplementary figure S2. (doi: 10.3897/zookeys.365.5728.app3) File format: Microsoft Word file (docx). [file ZooKeys-365-129-s003.docx]

**SI Figure S2.** One of most parsimonious trees with branch tips collapsed from the combined plastid and nuclear data

**
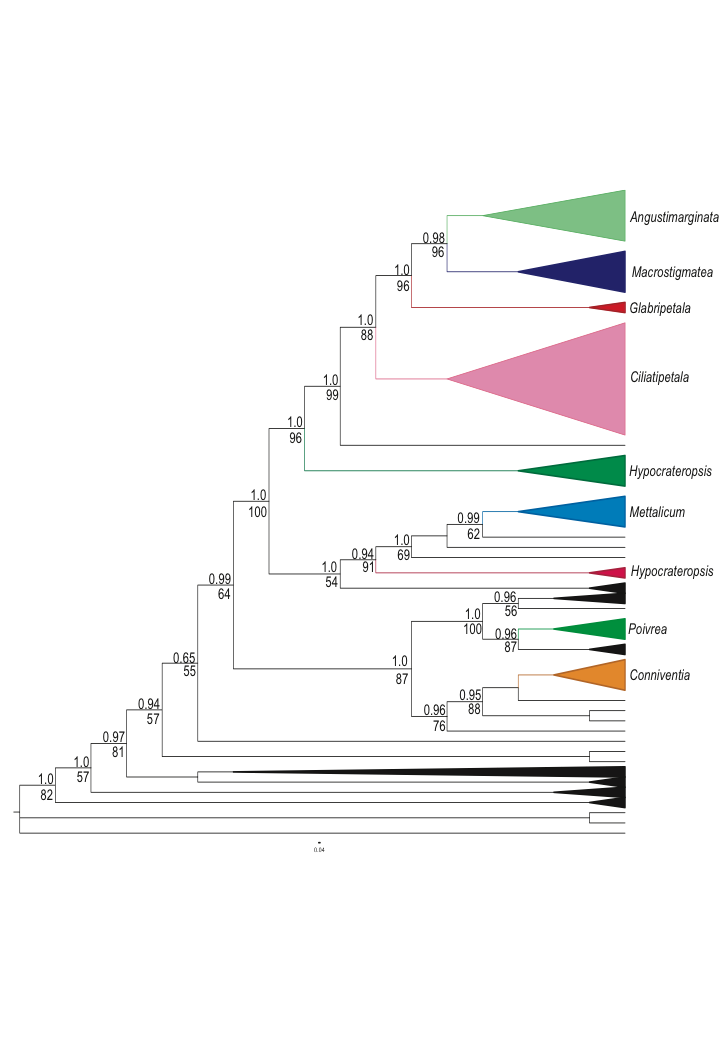
**
